# Time of Admission, Gender and Age: Challenging Factors in Emergency Renal Colic - A Preliminary Study

**DOI:** 10.5812/traumamon.6800

**Published:** 2012-10-10

**Authors:** Mohammad Javad Behzadnia, Hamid Reza Javadzadeh, Fatemeh Saboori

**Affiliations:** 1 Department of Emergency Medicine, Baqiyatallah University of Medical Sciences, Tehran, IR Iran

**Keywords:** Morphine, Renal Colic, Adverse Effect, Gender

## Abstract

**Background:**

Nephrolithiasis is a relatively common problem and a frequent Emergency Department (ED) diagnosis in patients who present with acute flank/abdominal pain. The pain management in these patients is often challenging.

**Objectives:**

To investigate the most effective dose of morphine with the least side effects in emergency renal colic patients.

**Materials and Methods:**

150 renal colic patients who experienced a pain level of 4 or greater, based on visual analog scale (VAS) at admission time were included. Pain was scored on a 100 mm VAS (0 = no pain, 100 = the worst pain imagined). When patients arrived at ED, a physician would examine the patients and assessed initial pain score, then filled a questionnaire according to the patient information. Patients were assigned to receive 2.5 mg morphine sulfate intravenously. We monitored patients’ visual analog scale (VAS), and adverse events at different time points (every 15 minutes) for 90 minutes. Additional doses of intravenous morphine (2.5 mg) were administered if the patient still had pain. (Max dose: 10 mg). The cumulative dose of morphine, defined as the total amount of morphine prescribed to each patient during the 90 minutes of the study, was recorded. Patients were not permitted to use any nonsteroidal anti-inflammatory drugs as coadjuvant analgesics during the study period. Subjects with inadequate pain relief at 90 minutes received rescue morphine and were excluded from the study. The primary end point in this study was pain relief at 90 minutes, defined as either VAS<40 or decrease of 50% or more as compared to the initial VAS. The secondary objective was to detect the occurrence of adverse effects at any time points in ED.

**Results:**

The studied patients consisted of 104 men and 46 women with the mean age of 43 ±14 years (range, 18 to 75 years). There was no statistically significant difference between the mean age and gender differences in pain response. Rescue analgesia at 30 minutes were given in 54.5% receiving morphine. The average time to painless was 35 minutes. But there were no statistically significant differences between the mean age and gender differences in pain response (P > 0.05). Older patients responded sooner to morphine than the young. Most of the patients had a pain score of 90 -100 (77.3 %) at the beginning that was reduced to 29.4% during the 30 minutes follow up. During the first hour, we found that 94.7% of the patients had no pain or significant pain reduction and only 2.1% of the patients still had pain.

**Conclusions:**

We conclude that there were no significant differences among the gender, time of admission and side - effects in renal colic patients in response to morphine.

## 1. Background

Pain is one of the most common reasons of emergency admission; it places burden on both patient and medical care system. Pain control is incomplete in most situations, especially in emergency settings. Pain management in emergency department (ED) is paramount (1-7). Nephrolithiasis is a relatively common problem and a frequent ED diagnosis in patients who present with acute flank/abdominal pain and pain management is a challenging task for physicians in this group. Acute renal colic is recognized by a sudden moderate to severe abdominal or flank pain that may refer to the genitalia. Most of the time patients cannot localize the pain. Nausea, vomiting and microscopic or macroscopic hematuria may be seen (8). Renal colic is seen in the patients 20- 40 years old in most of the studies and positive family history and inadequate water drinking are considered as risk factors (9). Its prevalence is different ranging from 5-15% in the American and European to 2-5 % in Asian population (10). In Iran, it was found that geographically, the west and north of Iran, and seasonally, in autumn showed the highest incidence rates for urinary tract stones (11). The classic treatment of non-complicated renal colic is hydration and proper pain control (12, 13). Opioids are considered as the cornerstone of moderate to severe pain control in ED. Opioid receptors are currently classified as mu (mu: mOP), delta (delta: dOP), kappa (kappa: kOP) with a fourth related non-classical opioid receptor for nociceptin/orphainin FQ, NOP (14). Opioid analgesics act by binding to special receptors in central and peripheral nervous system (7). Morphine is the current gold standard analgesic acting at MOP receptors but produces a range of variably troublesome adverse effects such as respiratory depression, pruritus, constipation, nausea, vomiting and urinary retention (14). Beside their side-effects, opioids have excellent analgesic properties in acute and chronic pains.

Theoretically, higher doses may lead to more unwanted effects especially in patients with severe pain. Thus titrated and appropriated dosage is important.

## 2. Objectives

There have been many trials aimed at reducing the side-effects of opioids; however, this study was designed to evaluate the effect of admission time and gender on side-effects of morphine sulfate, focusing on pain score in patients with renal colic in ED.

## 3. Materials and Methods

After receiving approval from the Ethical Board Committee of the Baqiyatallah Medical Sciences University (BMSU) and written informed consent, this study was conducted in the Emergency Department of the Baqiyatallah Hospital, one of the referral training hospitals in Tehran, I.R. Iran.

150 renal colic patients (18-75 years old) who experienced a pain level of 40 or higher, based on visual analog scale (VAS) at the admission time were included. Pain was scored on a 100 mm VAS (0 = no pain, 100 = the worst pain imagined). Patients were excluded from the study if they had allergy to study drugs, chronic opioid use, significant CNS myocardial, renal, or hepatic impairment, pyelonephritis or fever ≥ 38 degree centigrade, pregnancy and breast feeding and if they were hemodynamically unstable (BP ≤ 90 mmHg or MAP decreased ≥ 30% baseline) or any contraindications to anaesthesia. When patients arrived at the ED, a physician assessed the patients and initial pain score, and then filled a questionnaire according to the patient information. Data collected included age, sex, localization/severity of pain, symptoms, medical history and chemical lab tests and urine analysis in selected patients. Patients were assigned to receive 2.5 mg morphine sulfate intravenously. We monitored patients’ VAS and adverse events at different time points (every 15 minutes) for 90 minutes.

Additional doses of intravenous morphine (2.5 mg) were administered if the patient still had pain. (Max dose: 10 mg). The cumulative dose of morphine, defined as the total amount of morphine prescribed in each patient during the 90 minutes of the study, was recorded and used to compare the efficacy of the treatment in the 2 groups. Patients were not permitted to use any nonsteroidal antiinflammatory drugs as coadjuvant analgesics during the study period. Subjects with inadequate pain relief at 90 minutes received rescue morphine and were excluded from the study. The primary end point in this study was pain relief at 90 minutes, defined as either VAS < 40 or decrease of 50% or more as compared to the initial VAS. The secondary objective was to detect the occurrence of adverse effects at any time points in ED. Sample size was based on the power analysis from similar studies. Statistical analysis was performed using the Statistical Package for Social Sciences software (SPSS 17.0 for windows, SPSS Inc., IL, USA). Results were expressed as mean (SD), mean (range), median (inter-quartile range), or the number of patients. A P-value of less than 0.05 was considered statistically significant.

## 4. Results

**Figure 1:**
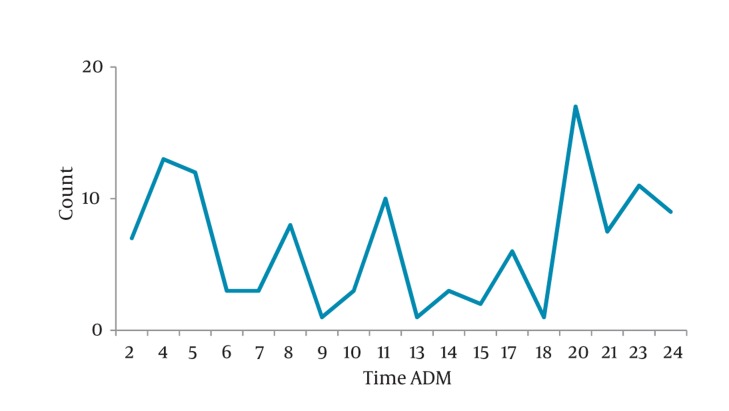
Admission Time Distribution (hour) of Renal Colic Patients

**Figure 2:**
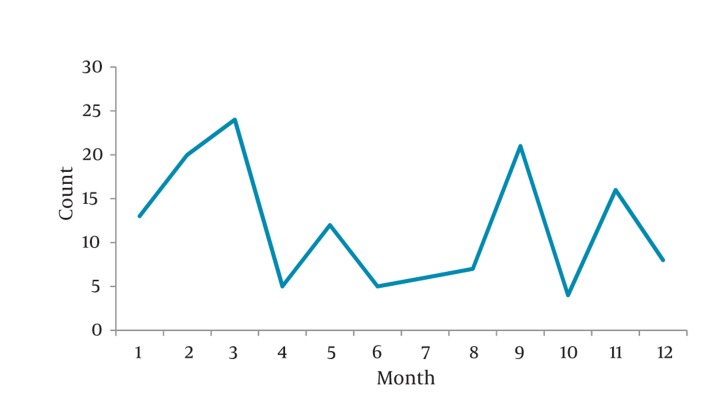
Admission Time Distribution (month) of Renal Colic Patients

The studied patients consisted of 104 men (69.3%) and 46 (30.7%) women with a mean age of 43 ± 14 years (range, 18 to 75 years). The peak of renal colic was seen during 7 p.m. to 5 a.m. Most of the cases came at : 4-5 a.m. (18.7%), 10-11 a.m. (6.7%) and 7-11 p.m. (34%) (Figure 1). Most renal colic patients were seen during in the first trimester of the Iranian year i.e., in the spring. The least cases were registered on the second half of year (Figure 2).

**Figure 3:**
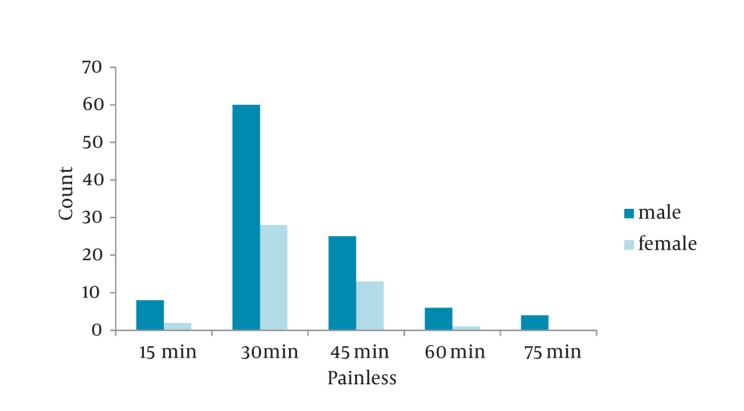
Painlessness Time Distribution (min) in Both the Genders

**Figure 4:**
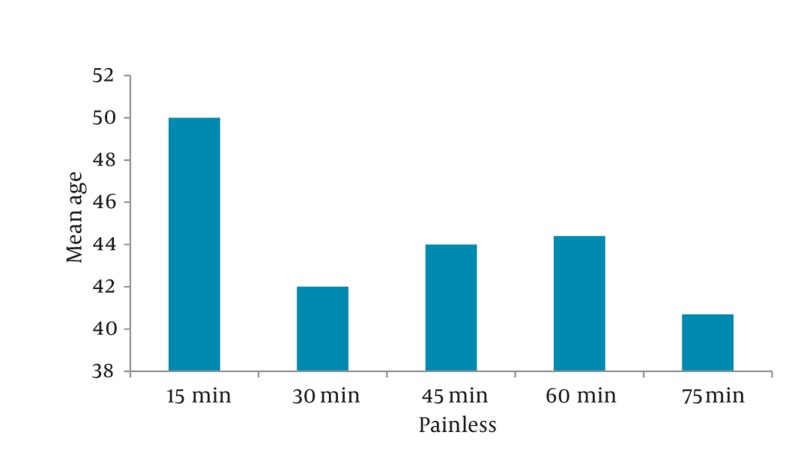
Painlessness Time Distribution (min) and Mean Age

Nausea (18.3%), nausea and vomiting (22.7%) were the most frequent side effects in ED. No patients in either group had respiratory depression and hypotension. No patients in either group had urinary retention during 90 minutes follow up in ED ; 108 subjects (36%) had a history of at least one episode of previous renal colic. Rescue analgesics at 30 minutes were needed in 54.5% receiving morphine. The average time to painlessness was 35 minutes (Figure 3). The older patients responded to morphine sooner than the younger patients (Figure 4).

In this study, most of the patients had a pain score of 90 -100 (77.3%) at the beginning that was reduced to 29.4% during the 30 minutes follow up. During the first hour, we found that 94.7% of the patients had no pain or significant pain reduction and only 2.1% of the studied population still had pain (Table 1).

**Table 1:** Frequency of Pain Scores (VAS) at Different Time Points

Time/Pain Score	10-9	8-7	6-5	4	3-1
**Pain at admission**	77.30 %	22.60 %	-	-	-
**Pain in 15 min**	72.60 %	20.70 %	-	-	6.70 %
**Pain in 30 min**	29.40 %	4 %	0.70 %	2 %	64 %
**Pain in 45 min**	5.40 %	1.30 %	-	-	93.30 %
**Pain in 60 min**	1.40 %	0.70 %	-	3.30 %	94.70 %
**Pain in 75 min**	-	-	-	4 %	96 %
**Pain in 90 min**	_	_	_	4 %	96 %

## 5. Discussion

Our study showed a male predominence (65%) in renal colic population compatible with similar studies. The age distribution was also similar to previous investigations (9). The peak of renal colic was seen during 7 p.m. to 5 a.m. Most of the cases came during the three time intervals: 4-5 a.m. (18.7%), 10-11 a.m. (6.7%) and 7-11 p.m. (34%) respectively. Maybe as cortisol level reduces during the day, and the pain perception arises specially at the end of the day time. Pruritus, nausea, and vomiting were the most common complications and also frequent symptoms in ED during the treatment in our study and several other previous studies (15). Sex differences in the analgesic effects of mu-opioid agonists has been documented extensively in animal and, to a lesser extent, in human studies (16). Female showed a much more reduction in pain score during 30- 45 minutes follow up that may contribute to different pain perception in different genders and also the role of opioid receptors characteristics in female due to hormonal effects (15, 17-21). Cepeda found that women had more intense pain and required 30% more morphine to achieve a similar degree of analgesia compared with men (22). There were no significant differences in side effects seen in this study and the similar ones (9). In this study, 64% of patients who received doses more than of 5 mg, became painless during the first 30 minutes. This result was confirmed by the other studies in which 60% did not want additional analgesics. The concentration of morphine used in this study was based on that used in other studies, which ranged from 0.055 mg/kg to 0.15 mg/kg (23-26).

## References

[1] Iyer SB, Schubert CJ, Schoettker PJ, Reeves SD (2011). Use of quality-improvement methods to improve timeliness of analgesic delivery.. Pediatrics.

[2] Morgan S (2011). Intravenous paracetamol in patients with renal colic.. Emerg Nurse.

[3] O'Connor AB, Zwemer FL, Hays DP, Feng C (2009). Outcomes after intravenous opioids in emergency patients: a prospective cohort analysis.. Acad Emerg Med.

[4] Raffa R (2006). Pharmacological aspects of successful long-term analgesia.. Clin Rheumatol.

[5] Steinberg PL, Nangia AK, Curtis K (2011). A standardized pain management protocol improves timeliness of analgesia among emergency department patients with renal colic.. Qual Manag Health Care.

[6] Tamches E, Buclin T, Hugli O, Decosterd I, Blanc C, Mouhsine E (2007). Acute pain in adults admitted to the emergency room: development and implementation of abbreviated guidelines.. Swiss Med Wkly.

[7] Wuhrman E, Cooney MF (2011). Acute Pain: Assessment and Treatment.. Topics in Advanced Practice Nursing e-journal.

[8] Eskelinen M, Ikonen J, Lipponen P (1998). Usefulness of history-taking, physical examination and diagnostic scoring in acute renal colic.. Eur Urol.

[9] Perez JA, Palmes Mde L, Ferrer JF, Urdangarain OO, Nunez AB (2010). Renal colic at emergency departments. Epidemiologic, diagnostic and etiopathogenic study.. Arch Esp Urol.

[10] Papa L, Stiell IG, Wells GA, Ball I, Battram E, Mahoney JE (2005). Predicting intervention in renal colic patients after emergency department evaluation.. CJEM.

[11] Basiri A, Shakhssalim N, Khoshdel AR, Mansouri M (2011). Seasonal variations of renal colics and urolithiasis: is this the time for a shared benchmark to study the phenomenon?. Urol Res.

[12] Cupisti A, Pasquali E, Lusso S, Carlino F, Orsitto E, Melandri R (2008). Renal colic in Pisa emergency department: epidemiology, diagnostics and treatment patterns.. Intern Emerg Med.

[13] Springhart WP, Marguet CG, Sur RL, Norris RD, Delvecchio FC, Young MD (2006). Forced versus minimal intravenous hydration in the management of acute renal colic: a randomized trial.. J Endourol.

[14] Dietis N, Guerrini R, Calo G, Salvadori S, Rowbotham DJ, Lambert DG (2009). Simultaneous targeting of multiple opioid receptors: a strategy to improve side-effect profile.. Br J Anaesth.

[15] Zun LS, Downey LV, Gossman W, Rosenbaumdagger J, Sussman G (2002). Gender differences in narcotic-induced emesis in the ED.. Am J Emerg Med.

[16] Comer SD, Cooper ZD, Kowalczyk WJ, Sullivan MA, Evans SM, Bisaga AM (2010). Evaluation of potential sex differences in the subjective and analgesic effects of morphine in normal, healthy volunteers.. Psychopharmacology (Berl).

[17] Banz VM, Christen B, Paul K, Martinolli L, Candinas D, Zimmermann H (2012). Gender, age and ethnic aspects of analgesia in acute abdominal pain: is analgesia even across the groups?. Intern Med J.

[18] Gear RW, Gordon NC, Miaskowski C, Paul SM, Heller PH, Levine JD (2003). Dose ratio is important in maximizing naloxone enhancement of nalbuphine analgesia in humans.. Neurosci Lett.

[19] Gear RW, Gordon NC, Miaskowski C, Paul SM, Heller PH, Levine JD (2003). Sexual dimorphism in very low dose nalbuphine postoperative analgesia.. Neurosci Lett..

[20] Li SF, Greenwald PW, Gennis P, Bijur PE, Gallagher EJ (2001). Effect of age on acute pain perception of a standardized stimulus in the emergency department.. Ann Emerg Med.

[21] Pud D, Yarnitsky D, Sprecher E, Rogowski Z, Adler R, Eisenberg E (2006). Can personality traits and gender predict the response to morphine? An experimental cold pain study.. Eur J Pain.

[22] Cepeda MS, Carr DB (2003). Women experience more pain and require more morphine than men to achieve a similar degree of analgesia.. Anesth Analg.

[23] O'Connor AB, Zwemer FL, Hays DP, Feng C (2010). Intravenous opioid dosing and outcomes in emergency patients: a prospective cohort analysis.. Am J Emerg Med.

[24] Aubrun F, Amour J, Rosenthal D, Coriat P, Riou B (2007). Effects of a loading dose of morphine before i.v. morphine titration for postoperative pain relief: a randomized, double-blind, placebo-control study.. Br J Anaesth.

[25] Galinski M, Dolveck F, Combes X, Limoges V, Smail N, Pommier V (2007). Management of severe acute pain in emergency settings: ketamine reduces morphine consumption.. Am J Emerg Med.

[26] Birnbaum A, Esses D, Bijur PE, Holden L, Gallagher EJ (2007). Randomized double-blind placebo-controlled trial of two intravenous morphine dosages (0.10 mg/kg and 0.15 mg/kg) in emergency department patients with moderate to severe acute pain.. Ann Emerg Med.

